# The Cell Death Inhibitor ARC Is Induced in a Tissue-Specific Manner by Deletion of the Tumor Suppressor Gene *Men1*, but Not Required for Tumor Development and Growth

**DOI:** 10.1371/journal.pone.0145792

**Published:** 2015-12-28

**Authors:** Wendy M. McKimpson, Ziqiang Yuan, Min Zheng, Judy S. Crabtree, Steven K. Libutti, Richard N. Kitsis

**Affiliations:** 1 Department of Medicine (Cardiology), Albert Einstein College of Medicine, Bronx, NY 10461, United States of America; 2 Department of Cell Biology, Albert Einstein College of Medicine, Bronx, NY 10461, United States of America; 3 Department of Surgery, Albert Einstein College of Medicine, Bronx, NY 10461, United States of America; 4 Department of Genetics, Albert Einstein College of Medicine, Bronx, NY 10461, United States of America; 5 Wilf Family Cardiovascular Research Institute, Albert Einstein College of Medicine, Bronx, NY 10461, United States of America; 6 Albert Einstein Cancer Center, Albert Einstein College of Medicine, Bronx, NY 10461, United States of America; 7 Einstein-Sinai Diabetes Research Center, Albert Einstein College of Medicine, Bronx, NY 10461, United States of America; 8 Department of Genetics, Louisiana State University Health Sciences Center, New Orleans, LA 70112, United States of America; Peking Union Medical College, CHINA

## Abstract

Multiple endocrine neoplasia type 1 (MEN1) is a genetic disorder characterized by tissue-specific tumors in the endocrine pancreas, parathyroid, and pituitary glands. Although tumor development in these tissues is dependent upon genetic inactivation of the tumor suppressor *Men1*, loss of both alleles of this gene is not sufficient to induce these cancers. *Men1* encodes menin, a nuclear protein that influences transcription. A previous ChIP on chip analysis suggested that menin binds promoter sequences of *nol3*, encoding ARC, which is a cell death inhibitor that has been implicated in cancer pathogenesis. We hypothesized that ARC functions as a co-factor with *Men1* loss to induce the tissue-restricted distribution of tumors seen in MEN1. Using mouse models that recapitulate this syndrome, we found that biallelic deletion of *Men1* results in selective induction of ARC expression in tissues that develop tumors. Specifically, loss of *Men1* in all cells of the pancreas resulted in marked increases in ARC mRNA and protein in the endocrine, but not exocrine, pancreas. Similarly, ARC expression increased in the parathyroid with inactivation of *Men1* in that tissue. To test if ARC contributes to MEN1 tumor development in the endocrine pancreas, we generated mice that lacked none, one, or both copies of ARC in the context of *Men1* deletion. Studies in a cohort of 126 mice demonstrated that, although mice lacking *Men1* developed insulinomas as expected, elimination of ARC in this context did not significantly alter tumor load. Cellular rates of proliferation and death in these tumors were also not perturbed in the absence of ARC. These results indicate that ARC is upregulated by loss *Men1* in the tissue-restricted distribution of MEN1 tumors, but that ARC is not required for tumor development in this syndrome.

## Introduction

Multiple endocrine neoplasia type 1 (MEN1) is a familial cancer syndrome characterized by tumors primarily in the endocrine pancreas, parathyroid gland, and anterior pituitary gland [1]. The genetic basis of MEN1 is loss of function mutations in the tumor suppressor gene *Men1*, with one mutant allele transmitted through the germ line and the other inactivated through somatic mutation [2–4]. *Men1* encodes menin, a nuclear protein [5] that regulates transcription, but whose precise biochemical function is not known [6].

As *Men1* is ubiquitously expressed [7], the basis for the highly tissue-restricted tumor distribution in MEN1 is not understood. The importance of *Men1* inactivation in the limited distribution of MEN1 tumors is demonstrated by the development of tumors in the endocrine pancreas, parathyroid, and pituitary of mice in which one allele has been deleted in the germline and the other inactivated by somatic mutation in those tissues [8, 9]. While differential loss of heterozygosity might contribute to the tissue-specific nature of MEN1 tumors, it is not sufficient to account for this phenomenon, as illustrated by the fact that forced deletion of both *Men1* alleles in certain tissues does not cause cancer. For example, *Men1* inactivation in the liver does not cause tumors in that organ [10], and deletion of *Men1* in all cells of the pancreas results in tumors in the endocrine, but not exocrine, tissues of this organ [11]. These observations suggest the existence of additional tissue-restricted factor(s) that cooperate with *Men1* loss in tumor development.

An important hallmark of cancer is the ability of transformed cells to evade apoptosis [12, 13]. Cancer cells often acquire the ability to suppress cell death through decreases in the abundance/activities of cell death promoters or increases in the analogous properties of cell death inhibitors such as Bcl-2 proteins [14], FLICE-like inhibitor protein (FLIP) [15], and inhibitor of apoptosis proteins (IAPs) [16]. Another cell death inhibitor that has recently been implicated in the pathogenesis of multiple cancers is Apoptosis Repressor with CARD (ARC). ARC inhibits cell death through its inactivation of both the extrinsic (death receptor) and intrinsic (mitochondrial/ER) apoptosis pathways. These actions are mediated through direct interactions of ARC with critical signaling components in these pathways [17–20].

In healthy mice, ARC is expressed primarily in cardiac and skeletal muscle [21], certain neuronal populations [22], and pancreatic islets, primarily β-cells [20]. However, ARC expression has been shown to be markedly induced in human malignancies and mouse models of cancer including breast [23, 24], colon [25], kidney [26], head and neck [27], and blood [28]. In human acute myelogenous leukemia, elevation of ARC levels correlate inversely with survival [28]. Further, deletion of *nol3*, which encodes ARC, in the context of a transgenic mouse model of breast cancer, ameliorates tumor growth, invasion, metastasis, and chemoresistance [29].

A previous ChIP on chip study suggesting that menin binds promoter sequences of *nol3* (NHGRI, GSE5357: Menin ChIP-chip) [30] led us to hypothesize that ARC may function as a co-factor in the tissue-restricted distribution of tumors in MEN1. Using mice in which *Men1* has been biallelically inactivated in specific tissues, we show that loss of *Men1* markedly induces ARC expression selectively in those tissues that are susceptible to MEN1 tumors. However, in contrast to its critical role in other cancers, elimination of ARC in the setting of *Men1* inactivation does not reduce β-cell tumor burden.

## Materials and Methods

### Mice

Pdx1-Cre; Men1 f/f mice [11] and PTH-Cre; Men1 f/f mice [31] have been described previously. We maintained the Pdx1-Cre transgene in a hemizygous state in all experiments. The Pdx1-Cre; Men1 f/f mice, initially on a mixed FVB/129Sv background, were backbred 3–4 generations onto C57BL/6 for these experiments. ARC -/- (gene name is *nol3*, but mice referred to as ARC -/- here for simplicity) mice on a C57BL/6 background are as previously described [32]. Studies were performed using mice of ages and genders indicated. Genetic controls were age- and gender-matched littermates. Studies were approved by the Institute for Animals Studies of the Albert Einstein College of Medicine.

### Laser capture microdissection (LCM)

Frozen sections (5 μM) from pancreatic and parathyroid tissues were stained using Arcturus HistoGene LCM Frozen Section Staining Kit (ThermoFisher Scientific). The stained tissue sections were microdissected using a Leica AS LMD Microsystem.

### qRT-PCR

Total RNA was extracted using RNeasy (Qiagen) and used to generate cDNA. qPCR was performed with a Gene Amp 7500 Sequence Detector (Applied Biosystems) using SYBR Green (Invitrogen) for quantification. Primer sequences to amplify ARC were 5’-CAAGAAGAGGATGAATCTGAAG-3’ (forward) and 5’-TTGGCAGTAGGTGTCTCG-3’ (reverse). Normalization was performed to 18S ribosomal RNA using primers 5’-GTAACCCGTTGAACCCCATT-3’ (forward) and 5’-CCATCCAATCGGTAGTAGCG-3’ (reverse).

### Immunoblotting

Islets were isolated from mice as previously described [20]. Approximately 200 islets (of similar size) were hand-picked for each mouse sample. Immunoblotting was performed as previously described [20]. Primary antibodies included ARC (Cayman) and α-tubulin (Sigma-Aldrich). IRDye 800 and 680 (LI-COR) were used to detect primary antibodies, and membranes scanned using a LI-COR Odyssey.

### FACS

Fresh pancreas tissue was digested for 30 min with Collagenase IV (0.8mg/ml) at 37°C. Following washing, cells were incubated for 20 min with primary antibodies against ARC (Cayman) and insulin (DAKO). The cells were then washed and incubated for 30 min with secondary antibodies Alexa Fluor 488 and 647 (Invitrogen) to detect ARC and insulin respectively. Following washing, cells were suspended in 200 μl of buffer and analyzed using a FACSAria cell sorter (BD Biosciences). The data were analyzed with CellQuest software (BD Biosciences).

### Metabolic Measurements

Blood glucose concentrations following an overnight fast were assayed using the OneTouch glucose monitoring system (LifeScan). Plasma insulin concentrations were measured by ELISA (ALPCO Diagnostics).

### Immunohistochemistry

Immunofluorescence was performed as previously described [20]. Pancreatic tissue was fixed in 10% neutral buffered formalin and antigen retrieval was completed with Antigen Unmasking Solution (Vector Laboratories). Primary antibodies included ARC (Cayman), Ki67 (Abcam), and insulin (Abcam), and secondary antibodies were Alexa Fluor 488 or 568 (Invitrogen). Samples were counterstained and coverslipped with VECTASHIELD HardSet Mounting Medium with DAPI (Vector Laboratories).

### Terminal deoxynucleotidyl transferase dUTP nick-end labeling (TUNEL)

Cell death was detected using the Fluorescein In Situ Cell Death Detection Kit (Roche Applied Science). Tissue sections were counterstained with insulin to demarcate pancreatic β-cells. A minimum of 5 fields per pancreas section was analyzed.

### Statistics

All data are presented as mean ± SEM. For comparisons between two groups, a two-tailed student’s t-test was performed. Multiple comparisons were analyzed using analysis of variance followed by a Tukey post-hoc test. Statistics were calculated using GraphPad Prism 5 software. P < 0.05 was deemed significant.

## Results

### Deletion of *Men1* increases expression of ARC preferentially in cell types that are susceptible to MEN1

**Fig 1:**
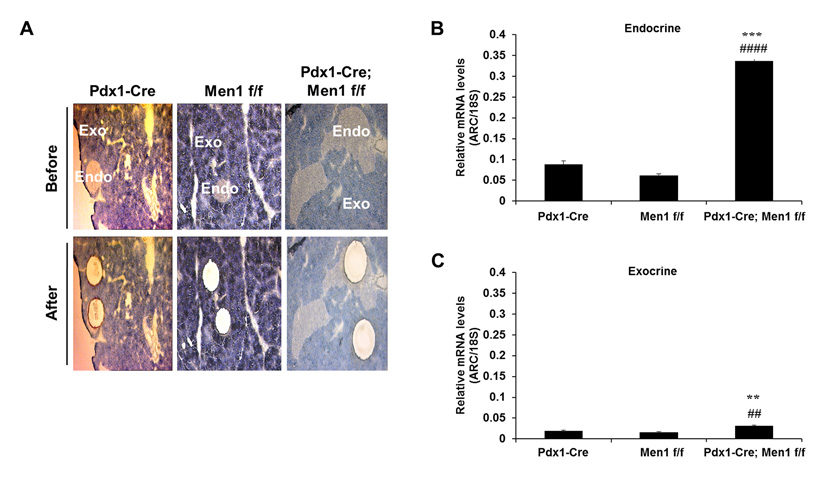
Deletion of *Men1* in all pancreatic cells increases ARC mRNA levels preferentially in islets. A) Endocrine (Endo) and exocrine (Exo) pancreatic tissue from 12 m old mice before (top) and after (bottom) laser capture microdissection. B) qRT-PCR for ARC in mouse pancreatic endocrine tissue from the indicated genotypes. C) qRT-PCR for ARC in mouse pancreatic exocrine tissue from the indicated genotypes. N = 3 mice per genotype. ** P < 0.01 versus Pdx1-Cre. *** P < 0.001 versus Pdx1-Cre, ^##^ P < 0.01 versus Men1 f/f, ^####^ P < 0.0001 versus Men1 f/f.

**Fig 2:**
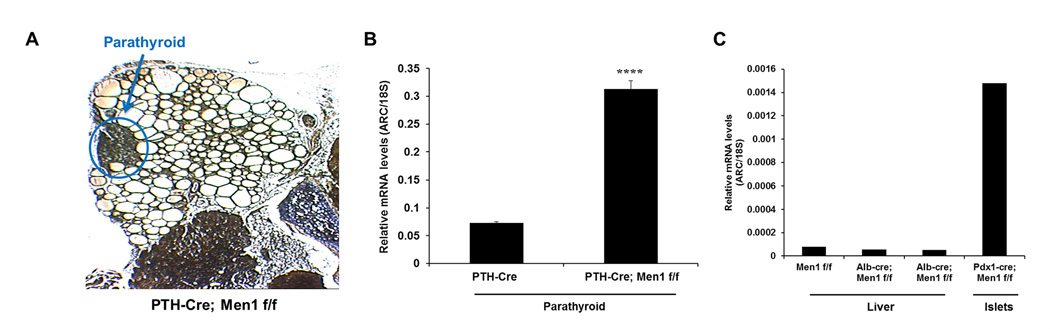
Deletion of *Men1* in the parathyroid hormone-secreting cells increases ARC mRNA expression. A) Parathyroid tissue (inside blue circle) from 12 m old PTH-Cre; Men1 f/f mouse subsequently subjected to laser microdissection. B) qRT-PCR for ARC in parathyroid tissue from the indicated genotypes. N = 3 mice per genotype. **** P < 0.0001 versus PTH-Cre. C) qRT-PCR for ARC in liver tissue from the indicated genotypes. Each bar in panel C represents mRNA samples isolated from a single mouse.

To assess whether ARC mediates the tissue-restricted nature of MEN1 tumors, we first asked whether the expression of ARC is induced by deletion of *Men1* selectively in those tissues that develop tumors in this syndrome. To address this, we used a Cre-loxP approach to delete *Men1* in cells of the endocrine pancreas (pancreatic islets), which develop MEN1 tumors, and then quantified the abundance of ARC transcripts specifically in this tissue using laser capture microdissection (Fig 1A). To provide an internal control, we chose to employ a Cre deletor (Pdx1-Cre), which would simultaneously excise *Men1* also in the exocrine pancreas, a tissue that does not develop MEN1 tumors. We observed that biallelic deletion of *Men1* in all cells of the pancreas resulted in marked increases in ARC expression in the endocrine pancreas (Fig 1B). In contrast, expression was increased only minimally in the exocrine tissue (Fig 1C). To further test the notion that loss of *Men1* increases ARC expression in tissues susceptible to MEN1 tumors, we deleted both alleles of *Men1* in the parathyroid gland using PTH-Cre, and again observed dramatic increases in the abundance of ARC mRNA (Fig 2A and 2B). In contrast, there were no changes in ARC expression when menin was deleted in the liver, which is consistent with this tissue not developing tumors in response to loss of *Men1* (Fig 2C).

**Fig 3:**
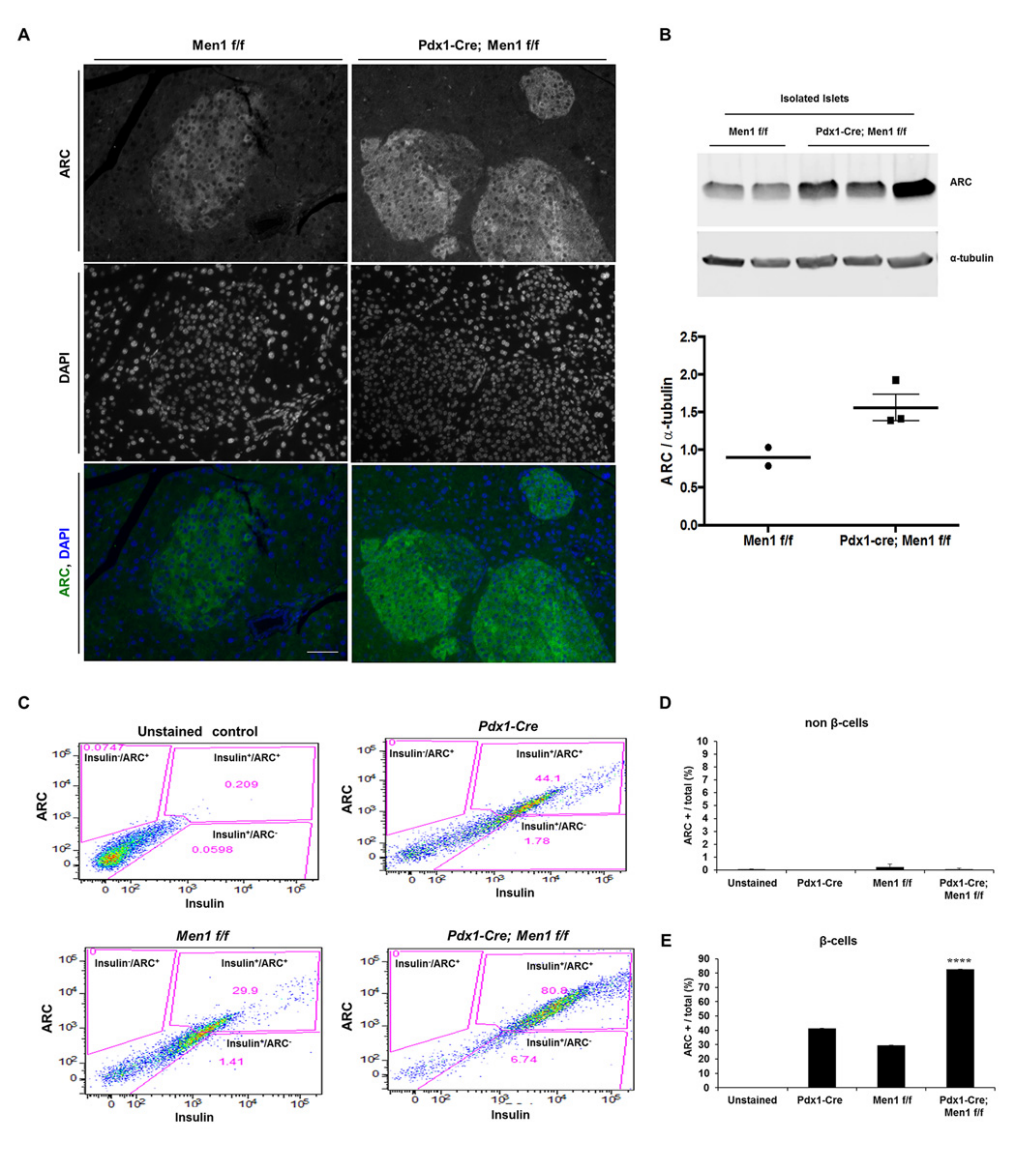
ARC protein is more abundant in β-cells of Pdx1-Cre; Men1 f/f mice. A) Immunostaining of pancreatic tissue for ARC from 12 m old mice. Nuclei counterstained with DAPI. Scale bar denotes 50 μM. B) Immunoblot (top) of islets isolated from Men1 f/f or Pdx1-Cre; Men1 f/f mice. Each lane represents islets isolated from an individual mouse. Quantification of ARC normalized to loading control (bottom). C) Representative FACS analysis of dissociated mouse pancreatic cells stained for ARC and insulin. D) Quantification of ARC-positive non-β-cells (as indicated by absence of insulin staining). E) Quantification of ARC-positive β-cells (insulin positive). N = 3 mice per genotype. **** P < 0.0001 versus each group.

To determine whether the increases in ARC mRNA in endocrine pancreas in response to *Men1* deletion resulted in increases in ARC protein, we first performed immunofluorescence. Consistent with our previous observations [20], ARC staining was restricted to islets and predominantly localized to the cytoplasm of these cells (Fig 3A). Importantly, under identical exposure conditions, ARC immunofluorescence was more prominent in tissue from Pdx1-Cre; Men1 f/f, as compared with Men1 f/f, mice (compare Fig 3A left and right). These increases in ARC protein were quantified using immunoblotting of isolated islets (Fig 3B). We also analyzed cells dissociated from whole pancreas tissue using FACS (Fig 3C–3E). The percentage of ARC-positive β-cells (insulin-positive) was 2–3-fold greater in pancreata from Pdx1-Cre; Men1 f/f, as compared with Men1 f/f, mice. These three approaches, in conjunction with the qRT-PCR data in Fig 1, demonstrate that deletion of *Men1* results in increased ARC mRNA and protein selectively in β-cells.

### Genetic elimination of ARC does not reverse the development of insulinomas resulting from inactivation of *Men1*

The induction of ARC expression selectively in tissues that develop MEN1 tumors raises the possibility that ARC may cooperate with *Men1* loss to promote tissue-restricted tumors. To test the role of ARC in the development of insulinomas, we bred Pdx1-Cre; Men1 f/f mice with mice in which the gene encoding ARC had been deleted in the germ line to create mice lacking both menin and ARC in all cells of the pancreas. Although ARC -/- mice have no basal phenotype, we hypothesized that the absence of ARC might ameliorate the development of tumors resulting from deletion of *Men1*.

**Fig 4:**
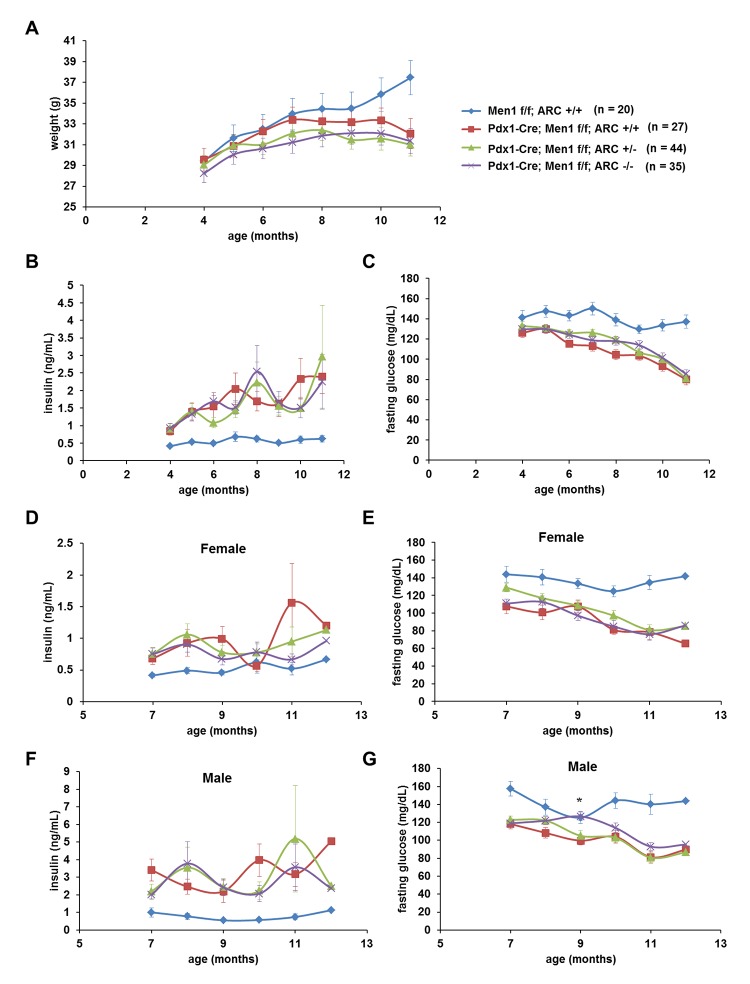
Generalized deletion of ARC in Pdx1-Cre; Men1 f/f mice does not significantly change islet tumor load. A) Body weights (both genders). B) Fasting plasma insulin concentrations (both genders). C) Fasting blood glucose concentrations (both genders). D) and E) Fasting insulin and glucose measurements in females. F) and G) Fasting insulin and glucose measurements in males. * P < 0.05 Pdx1-Cre; Men1 f/f; ARC -/- versus Pdx1-Cre; Men1 f/f; ARC +/+.

**Fig 5:**
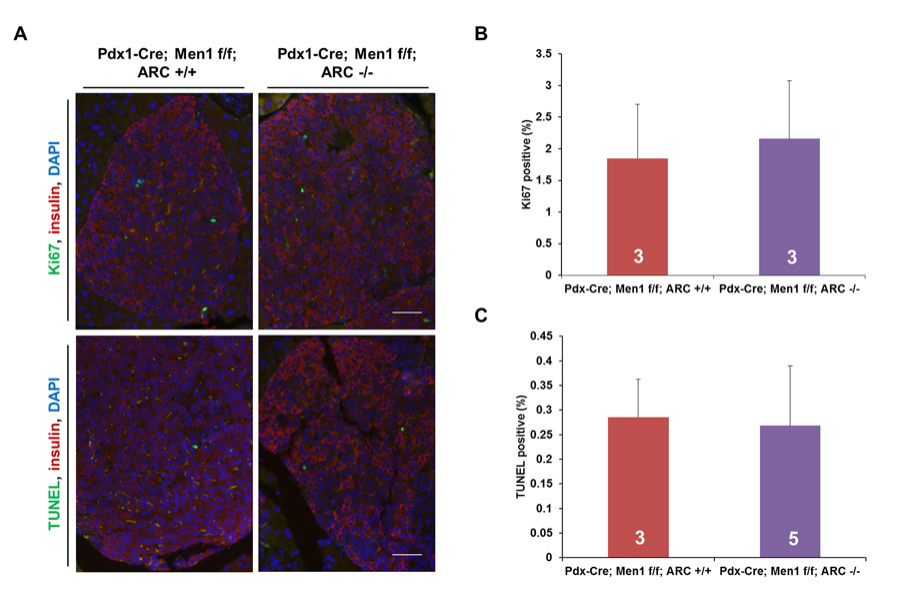
Deletion of ARC does not change rates of cellular proliferation and apoptosis in MEN1 insulinomas. A) Immunostaining of pancreatic tissue from 12 m old mice for Ki-67 (top, green) and TUNEL (bottom, green). β-cells are demarcated by insulin (red) and nuclei are counterstained with DAPI. Scale bar denotes 50 μM. B) Quantification of proliferating cells by Ki-67 staining. C) Quantification of apoptotic cells by TUNEL. No comparisons are significant. Number of mice in each group as indicated.

Biallelic deletion of *Men1* in the pancreas results in β-cells tumors accompanied by blunting of gains in body weight (Fig 4A) [11, 33, 34]. The tumor burden from these insulinomas is quantified clinically and in experimental models using plasma insulin concentrations [11]. As expected, we observed age-dependent increases in plasma insulin levels in Pdx1-Cre; Men1 f/f mice compared with Men1 f/f mice (Fig 4B, red curve compared with blue curve). Since insulin stimulates the uptake of glucose into cells, Pdx1-Cre; Men1 f/f mice also exhibited reciprocal decreases in fasting blood glucose concentrations (Fig 4C). Deletion of either one or both alleles encoding ARC did not normalize insulin or glucose levels, even in the large cohort of mice with 20–44 mice of each genotype (Fig 4B and 4C, green and purple curves compared with red curve). Similarly, loss of ARC had no significant effects on tumor burden when female and male mice were analyzed individually (Fig 4D–4G). To further analyze the role of ARC on β-cell tumor load, we assessed the effects of ARC deletion on β-cell proliferation and apoptosis in Pdx1-Cre; Men1 f/f mice (Fig 5A). Loss of ARC did not affect either parameter (Fig 5B and 5C). We conclude that ARC does not appear critical in the pathogenesis of insulinomas in this mouse model of MEN1.

## Discussion

The mechanistic basis by which *Men1* inactivation increases ARC expression preferentially in tissues that develop MEN1 tumors is not known. While changes in ARC protein abundance may be regulated through both transcriptional mechanisms [35, 36] and at the level of protein stability [37–39], here increases in mRNA levels appear adequate to account for increases in ARC protein. Menin is a nuclear protein [5], and a previous ChIP-chip analysis suggests that it binds promoter sequences of *nol3*, which encodes ARC (NHGRI, GSE5357: Menin ChIP-chip) [30]. Thus, consistent with data suggesting that menin may function as a transcription factor [40, 41], one potential mechanism for increases in ARC mRNA following deletion of *Men1* is that menin directly represses *nol3* transcription. Other data, however, suggest that menin modulates transcription indirectly through its interactions with transcription factors, including both activators and repressors [6], and/or through promotion of epigenetic modification of histones [40, 42]. Therefore, another possible mechanism is that menin decreases ARC transcription through these mechanisms, although it is not currently known whether any of the transcription factors that menin is known to bind/modify regulate *nol3* transcription. Another indirect mechanism by which menin may regulate *nol3* transcription is through its established inhibition of ERK signaling [43], as MEK/ERK is known to mediate increases in *nol3* transcription [35, 44]. The basis underlying the tissue-specificity of the increases in ARC expression following loss of menin is also elusive and could, in theory, be related to differences in the chromatin state of the *nol3* locus in various tissues or to any of the indirect mechanisms discussed above. For example, menin might repress *nol3* transcription through inhibition of a transcriptional activator whose presence is restricted to tumor-susceptible tissues.

Interestingly, we found that the increases in ARC levels resulting from *Men1* inactivation were not required for the development of MEN1 tumors. ARC abundance increases in multiple human cancers [23–28], and the absence of an effect of *nol3* deletion on MEN1 tumor load in this study contrasts with the amelioration of tumor growth (as well as metastasis) resulting from *nol3* inactivation in a genetic mouse model of breast cancer [29]. While the absence of an effect could reflect redundancy from another cell death inhibitor, a more likely possibility is differences in the cancer biology of MEN1 tumors in comparison to breast cancers. In the breast cancer model, the major effect of *nol3* deletion on tumor growth was to reduce rates of tumor cell proliferation (mechanism not known), while basal rates of apoptosis were not affected [29]. In contrast, *nol3* deletion affected neither basal rates of cell proliferation nor cell death in MEN1 insulinomas, which likely accounts for the absence of an effect of loss of ARC in these tumors.

Since ARC inhibits apoptosis in both transformed β-cells [20] and breast cancer cells [23], why did *nol3* deletion not affect rates of apoptosis in insulinomas and breast tumors? One possibility is that basal rates of cell death are relatively low in both of these tumors. Consistent with this hypothesis, *nol3* deletion markedly exacerbates apoptosis when mice harboring breast tumors are treated with chemotherapeutic agents [29]. Whether MEN1 tumors would behave similarly remains to be determined. Second, the ability of ARC to suppress basal levels of apoptosis may be dependent on the nature of the inciting death stimuli. For example, we have shown that ARC potently inhibits apoptosis in β-cells stimulated with ER stressors [20], but the precise nature of the death stimuli that operate in insulinomas is not known.

## References

[1] Friedman E. LC, Amorosi A., Brandi M.L., Bale A., Metz D., Jensen R.T., Skarulis M., Eastman R.C., Nieman L., Norton J.A., Marx S.J.. Multiple endocrine neoplasia type 1: Pathology, pathophysiology, and differential diagnosis In: Bilezekian J.P. LMA, Marcus R., editor. The Parathyroids: Raven Press; 1994 p. 647–80.

[2] Bassett JH, Forbes SA, Pannett AA, Lloyd SE, Christie PT, Wooding C, et al Characterization of mutations in patients with multiple endocrine neoplasia type 1. American journal of human genetics. 1998;62(2):232–44. 10.1086/301729 9463336 PMC1376903

[3] Chandrasekharappa SC, Guru SC, Manickam P, Olufemi SE, Collins FS, Emmert-Buck MR, et al Positional cloning of the gene for multiple endocrine neoplasia-type 1. Science. 1997;276(5311):404–7. . 9103196 10.1126/science.276.5311.404

[4] Larsson C, Skogseid B, Oberg K, Nakamura Y, Nordenskjold M. Multiple endocrine neoplasia type 1 gene maps to chromosome 11 and is lost in insulinoma. Nature. 1988;332(6159):85–7. 10.1038/332085a0 . 2894610

[5] Guru SC, Goldsmith PK, Burns AL, Marx SJ, Spiegel AM, Collins FS, et al Menin, the product of the MEN1 gene, is a nuclear protein. Proceedings of the National Academy of Sciences of the United States of America. 1998;95(4):1630–4. 9465067 10.1073/pnas.95.4.1630 PMC19125

[6] Matkar S, Thiel A, Hua X. Menin: a scaffold protein that controls gene expression and cell signaling. Trends in biochemical sciences. 2013;38(8):394–402. 10.1016/j.tibs.2013.05.005 23850066 PMC3741089

[7] Stewart C, Parente F, Piehl F, Farnebo F, Quincey D, Silins G, et al Characterization of the mouse Men1 gene and its expression during development. Oncogene. 1998;17(19):2485–93. 10.1038/sj.onc.1202164 . 9824159

[8] Crabtree JS, Scacheri PC, Ward JM, Garrett-Beal L, Emmert-Buck MR, Edgemon KA, et al A mouse model of multiple endocrine neoplasia, type 1, develops multiple endocrine tumors. Proceedings of the National Academy of Sciences of the United States of America. 2001;98(3):1118–23. 10.1073/pnas.98.3.1118 11158604 PMC14718

[9] Bertolino P, Tong WM, Galendo D, Wang ZQ, Zhang CX. Heterozygous Men1 mutant mice develop a range of endocrine tumors mimicking multiple endocrine neoplasia type 1. Molecular endocrinology. 2003;17(9):1880–92. 10.1210/me.2003-0154 . 12819299

[10] Scacheri PC, Crabtree JS, Kennedy AL, Swain GP, Ward JM, Marx SJ, et al Homozygous loss of menin is well tolerated in liver, a tissue not affected in MEN1. Mammalian genome: official journal of the International Mammalian Genome Society. 2004;15(11):872–7. . 15672591 10.1007/s00335-004-2395-z

[11] Shen HC, He M, Powell A, Adem A, Lorang D, Heller C, et al Recapitulation of pancreatic neuroendocrine tumors in human multiple endocrine neoplasia type I syndrome via Pdx1-directed inactivation of Men1. Cancer research. 2009;69(5):1858–66. 10.1158/0008-5472.CAN-08-3662 19208834 PMC3879686

[12] Hanahan D, Weinberg RA. The hallmarks of cancer. Cell. 2000;100(1):57–70. . 10647931 10.1016/s0092-8674(00)81683-9

[13] Hanahan D, Weinberg RA. Hallmarks of cancer: the next generation. Cell. 2011;144(5):646–74. 10.1016/j.cell.2011.02.013 . 21376230

[14] Yip KW, Reed JC. Bcl-2 family proteins and cancer. Oncogene. 2008;27(50):6398–406. 10.1038/onc.2008.307 . 18955968

[15] Subramaniam K, Hirpara JL, Tucker-Kellogg L, Tucker-Kellogg G, Pervaiz S. FLIP: a flop for execution signals. Cancer letters. 2013;332(2):151–5. 10.1016/j.canlet.2012.07.005 . 22781394

[16] Hunter AM, LaCasse EC, Korneluk RG. The inhibitors of apoptosis (IAPs) as cancer targets. Apoptosis: an international journal on programmed cell death. 2007;12(9):1543–68. 10.1007/s10495-007-0087-3 . 17573556

[17] Nam YJ, Mani K, Ashton AW, Peng CF, Krishnamurthy B, Hayakawa Y, et al Inhibition of both the extrinsic and intrinsic death pathways through nonhomotypic death-fold interactions. Mol Cell. 2004;15(6):901–12. Epub 2004/09/24. 10.1016/j.molcel.2004.08.020 . 15383280

[18] Gustafsson AB, Tsai JG, Logue SE, Crow MT, Gottlieb RA. Apoptosis repressor with caspase recruitment domain protects against cell death by interfering with Bax activation. J Biol Chem. 2004;279(20):21233–8. Epub 2004/03/09. 10.1074/jbc.M400695200 . 15004034

[19] Foo RS, Nam YJ, Ostreicher MJ, Metzl MD, Whelan RS, Peng CF, et al Regulation of p53 tetramerization and nuclear export by ARC. Proceedings of the National Academy of Sciences of the United States of America. 2007;104(52):20826–31. 10.1073/pnas.0710017104 18087040 PMC2409226

[20] McKimpson WM, Weinberger J, Czerski L, Zheng M, Crow MT, Pessin JE, et al The apoptosis inhibitor ARC alleviates the ER stress response to promote beta-cell survival. Diabetes. 2013;62(1):183–93. 10.2337/db12-0504 22933109 PMC3526036

[21] Koseki T, Inohara N, Chen S, Nunez G. ARC, an inhibitor of apoptosis expressed in skeletal muscle and heart that interacts selectively with caspases. Proceedings of the National Academy of Sciences of the United States of America. 1998;95(9):5156–60. 9560245 10.1073/pnas.95.9.5156 PMC20230

[22] Geertman R, McMahon A, Sabban EL. Cloning and characterization of cDNAs for novel proteins with glutamic acid-proline dipeptide tandem repeats. Biochimica et biophysica acta. 1996;1306(2–3):147–52. . 8634331 10.1016/0167-4781(96)00036-x

[23] Mercier I, Vuolo M, Madan R, Xue X, Levalley AJ, Ashton AW, et al ARC, an apoptosis suppressor limited to terminally differentiated cells, is induced in human breast cancer and confers chemo- and radiation-resistance. Cell Death Differ. 2005;12(6):682–6. Epub 2005/04/30. 10.1038/sj.cdd.4401631 . 15861191

[24] Wang M, Qanungo S, Crow MT, Watanabe M, Nieminen AL. Apoptosis repressor with caspase recruitment domain (ARC) is expressed in cancer cells and localizes to nuclei. FEBS letters. 2005;579(11):2411–5. 10.1016/j.febslet.2005.03.040 . 15848180

[25] Mercier I, Vuolo M, Jasmin JF, Medina CM, Williams M, Mariadason JM, et al ARC (apoptosis repressor with caspase recruitment domain) is a novel marker of human colon cancer. Cell Cycle. 2008;7(11):1640–7. Epub 2008/05/13. . 18469522 10.4161/cc.7.11.5979

[26] Heikaus S, Kempf T, Mahotka C, Gabbert HE, Ramp U. Caspase-8 and its inhibitors in RCCs in vivo: the prominent role of ARC. Apoptosis: an international journal on programmed cell death. 2008;13(7):938–49. 10.1007/s10495-008-0225-6 . 18516683

[27] Wu P, Tang Y, He J, Qi L, Jiang W, Zhao S. ARC is highly expressed in nasopharyngeal carcinoma and confers X-radiation and cisplatin resistance. Oncology reports. 2013;30(4):1807–13. 10.3892/or.2013.2622 . 23877130

[28] Carter BZ, Qiu YH, Zhang N, Coombes KR, Mak DH, Thomas DA, et al Expression of ARC (apoptosis repressor with caspase recruitment domain), an antiapoptotic protein, is strongly prognostic in AML. Blood. 2011;117(3):780–7. 10.1182/blood-2010-04-280503 21041716 PMC3035072

[29] Medina-Ramirez CM, Goswami S, Smirnova T, Bamira D, Benson B, Ferrick N, et al Apoptosis inhibitor ARC promotes breast tumorigenesis, metastasis, and chemoresistance. Cancer Res. 2011;71(24):7705–15. Epub 2011/11/01. 10.1158/0008-5472.CAN-11-2192 22037876 PMC3245742

[30] Scacheri PC, Davis S, Odom DT, Crawford GE, Perkins S, Halawi MJ, et al Genome-wide analysis of menin binding provides insights into MEN1 tumorigenesis. PLoS genetics. 2006;2(4):e51 10.1371/journal.pgen.0020051 16604156 PMC1428788

[31] Libutti SK, Crabtree JS, Lorang D, Burns AL, Mazzanti C, Hewitt SM, et al Parathyroid gland-specific deletion of the mouse Men1 gene results in parathyroid neoplasia and hypercalcemic hyperparathyroidism. Cancer research. 2003;63(22):8022–8. . 14633735

[32] Zaiman AL, Damico R, Thoms-Chesley A, Files DC, Kesari P, Johnston L, et al A critical role for the protein apoptosis repressor with caspase recruitment domain in hypoxia-induced pulmonary hypertension. Circulation. 2011;124(23):2533–42. Epub 2011/11/16. 10.1161/CIRCULATIONAHA.111.034512 . 22082675 PMC4583146

[33] Bertolino P, Tong WM, Herrera PL, Casse H, Zhang CX, Wang ZQ. Pancreatic beta-cell-specific ablation of the multiple endocrine neoplasia type 1 (MEN1) gene causes full penetrance of insulinoma development in mice. Cancer research. 2003;63(16):4836–41. . 12941803

[34] Crabtree JS, Scacheri PC, Ward JM, McNally SR, Swain GP, Montagna C, et al Of mice and MEN1: Insulinomas in a conditional mouse knockout. Molecular and cellular biology. 2003;23(17):6075–85. 12917331 10.1128/MCB.23.17.6075-6085.2003 PMC180910

[35] Wu L, Nam YJ, Kung G, Crow MT, Kitsis RN. Induction of the apoptosis inhibitor ARC by Ras in human cancers. J Biol Chem. 2010;285(25):19235–45. Epub 2010/04/16. 10.1074/jbc.M110.114892 20392691 PMC2885202

[36] Li YZ, Lu DY, Tan WQ, Wang JX, Li PF. p53 initiates apoptosis by transcriptionally targeting the antiapoptotic protein ARC. Molecular and cellular biology. 2008;28(2):564–74. 10.1128/MCB.00738-07 17998337 PMC2223427

[37] Foo RS, Chan LK, Kitsis RN, Bennett MR. Ubiquitination and degradation of the anti-apoptotic protein ARC by MDM2. J Biol Chem. 2007;282(8):5529–35. Epub 2006/12/05. 10.1074/jbc.M609046200 . 17142834

[38] Nam YJ, Mani K, Wu L, Peng CF, Calvert JW, Foo RS, et al The apoptosis inhibitor ARC undergoes ubiquitin-proteasomal-mediated degradation in response to death stimuli: identification of a degradation-resistant mutant. J Biol Chem. 2007;282(8):5522–8. Epub 2006/12/05. 10.1074/jbc.M609186200 . 17142452

[39] Li J, Li C, Zhang D, Shi D, Qi M, Feng J, et al SNX13 reduction mediates heart failure through degradative sorting of apoptosis repressor with caspase recruitment domain. Nature communications. 2014;5:5177 10.1038/ncomms6177 . 25295779

[40] Karnik SK, Hughes CM, Gu X, Rozenblatt-Rosen O, McLean GW, Xiong Y, et al Menin regulates pancreatic islet growth by promoting histone methylation and expression of genes encoding p27Kip1 and p18INK4c. Proceedings of the National Academy of Sciences of the United States of America. 2005;102(41):14659–64. 10.1073/pnas.0503484102 16195383 PMC1253549

[41] Milne TA, Hughes CM, Lloyd R, Yang Z, Rozenblatt-Rosen O, Dou Y, et al Menin and MLL cooperatively regulate expression of cyclin-dependent kinase inhibitors. Proceedings of the National Academy of Sciences of the United States of America. 2005;102(3):749–54. 10.1073/pnas.0408836102 15640349 PMC545577

[42] Lin W, Watanabe H, Peng S, Francis JM, Kaplan N, Pedamallu CS, et al Dynamic epigenetic regulation by menin during pancreatic islet tumor formation. Molecular cancer research: MCR. 2015;13(4):689–98. 10.1158/1541-7786.MCR-14-0457 . 25537453

[43] Gallo A, Cuozzo C, Esposito I, Maggiolini M, Bonofiglio D, Vivacqua A, et al Menin uncouples Elk-1, JunD and c-Jun phosphorylation from MAP kinase activation. Oncogene. 2002;21(42):6434–45. 10.1038/sj.onc.1205822 . 12226747

[44] Mak PY, Mak DH, Ruvolo V, Jacamo R, Kornblau SM, Kantarjian H, et al Apoptosis repressor with caspase recruitment domain modulates second mitochondrial-derived activator of caspases mimetic-induced cell death through BIRC2/MAP3K14 signalling in acute myeloid leukaemia. British journal of haematology. 2014;167(3):376–84. 10.1111/bjh.13054 25079338 PMC4357400

